# An open dataset on individual perceptions of transport policies

**DOI:** 10.1038/s41597-024-02950-9

**Published:** 2024-01-22

**Authors:** Minh Kieu, Alexis Comber, Hang Nguyen Thi Thuy, Thanh Bui Quang, Phe Hoang Huu, Nick Malleson

**Affiliations:** 1 Department of Civil and Environmental Engineering, University of Auckland, Auckland, 1010 New Zealand; 2 School of Geography, University of Leeds, LS2 9JT Leeds, UK; 3 Faculty of Geography, VNU University of Science, Hanoi, Vietnam; 4 R&D Consultants, Hanoi, Vietnam; 5 Alan Turing Institute, NW1 2DB London, UK

**Keywords:** Geography, Developing world

## Abstract

Many cities are facing challenges caused by the increasing use of motorised transport and Hanoi, Vietnam, is no exception. The proliferation of petrol powered motorbikes has caused serious problems of congestion, pollution, and road safety. This paper reports on a new survey dataset that was created as part of the Urban Transport Modelling for Sustainable Well-Being in Hanoi (UTM-Hanoi) project. The survey of nearly 30,000 respondents gathers data on households’ demographics, perceptions, opinions and stated behaviours. The data are informative in their own right and have also been used to experiment with multi-scale spatial statistics, synthetic population generation and machine learning approaches to predicting an individual’s perceptions of potential government policies. The paper reports on the key findings from the survey and conducts a technical validation to contrast the outcomes to similar datasets that are available.

## Background & Summary

Increases in wealth and motorisation in numerous developing countries have led to a considerable escalation in traffic congestion. Hanoi, the capital of Vietnam, exemplifies this transformation. Motorbikes have emerged as an affordable, convenient, and fuel-efficient mode of transportation in this densely populated city, with the added advantage of easy parking in narrow streets and alleys^1^. However, they have transformed Hanoi from a serene city abundant with bicycles to a bustling, polluted metropolis teeming with petrol motorbikes, characterised by incessant buzzing and honking^2^.

The metamorphosis of Hanoi’s automobile landscape is inextricably linked to the city’s political climate. The 1986 economic reforms known as Doi Moi opened Vietnam to regional and global capitalism, ultimately transforming urban mobility^3^. The shift towards a market economy and the liberalisation of private acquisition catalysed the transition from bicycle-dominated streets to motorbike-centric transportation. Doi Moi spurred rapid urbanisation, introducing new leisure activities, employment opportunities, and housing options, which in turn increased the demand for intra-urban and urban-rural mobility^4^. Hanoi’s streetscape, originally designed for bicycles with its intricate network of alleyways and narrow roads, proved to be ideally suited to motorbikes. This mode of transportation effectively addressed the burgeoning mobility requirements in the wake of poor public transport infrastructure and services. Between 1996 and 2014, the number of motorbikes in Vietnam increased tenfold, from 4 million to 43 million, with 5 million motorbikes in Hanoi alone, equating to two and a half motorbikes per household^5^.

The proliferation of motorbikes has caused traffic congestion, an increase in the number of fatal vehicle accidents and serious environmental problems such as air and noise pollution^1,2,6–10^. For these reasons, the Vietnamese government has made plans to transition away from motorbikes and towards public transportation as the predominant modal choice, including a proposed ban on motorbikes in the city of Hanoi in 2030^11^. However, a potential ban of motorbikes will be controversial in Hanoi, as many households are still rely on them for mobility^3,7,12^. Despite the recent investments in public transport, the current transit system in Hanoi is very limited^13^ and many motorbike users are considering upgrading to cars^1^.

Nevertheless, it is challenging to anticipate public opinion on radical transport policies, such as the motorbike ban in Hanoi. Policymakers must understand the overall transport system itself, as well as public opinions and perceptions of transport policies, to make informed decisions. In matters of transportation and land use planning there is a well-known tendency towards the status quo. Transportation surveys and opinion polls allow governments to gauge the popularity, necessity, and viability of future policies and are a vital input in the policy making process. Policies that were constructed without thorough public consultation are often deemed to be inadequate for meeting the public’s needs^14,15^.

This article introduces a new data set that was compiled from the results of an individual travel and perception survey collected as part of the “Urban Transport Modelling for Sustainable Well-Being in Hanoi” (UTM-Hanoi) project. The data set is a unique survey of citizens’ demographics, perceptions, opinions and stated behaviours in Hanoi. The overarching goal of UTM-Hanoi is to develop data-driven and equitable transportation policies through close collaboration with government stakeholders in Hanoi. The survey, which has resulted in the UTM-Hanoi dataset presented in this manuscript, aims at quantifying public perceptions towards transportation modes and policies, especially on a potential ban of motorcycles in central districts of Hanoi. Despite concerns over feasibility and public acceptance, Hanoi authorities issued Decision 5953 to ban motorbikes in certain areas by 2030 as part of a broader plan to reduce motorbike usage and increase public transit ridership. The decision proposes gradually prohibiting non-electric motorcycles while raising the public transit mode share to 65%^16^. This controversial policy sparked many unanswered questions about mobility impacts on most Hanoi residents, such as whether public transit can adequately meet travel needs or if better alternatives exist.

Earlier versions of the dataset have been used to experiment with multi-scale spatial statistics^17,18^, synthetic population generation^19^ and machine learning approaches to predicting an individual’s perceptions of potential government policies^20^. However, the dataset described in this paper is the only complete dataset, with the total of 26,106 households (^17^ and^19^ used a sample of around 1500 households), with all variables included (both^18^ and^20^ focus on 10–15 variables that are significant to their models).

The dataset described in this paper has 56 variables within the 3 categories of questionnaires: “General info”, “Transport behaviour”, and “Perceptions on transport policies”. This dataset provides a comprehensive view of Hanoi’s urban transport landscape from the perspective of its residents. Besides the objectives related to transport patterns and prefenrences, such as in^18^ and^20^, other variables also can facilitate further understanding of many different purposes, such as the quality of life in Hanoi, the perceptions on transportation modes or the distribution of socio-demographics to different spatial areas in the city.

## Methods

### Aims and motivation

UTM-Hanoi is a large-scale travel survey data that can enable us to better understand the transportation landscape in Hanoi and inform the development of sustainable and effective urban transportation policies. The travel survey dataset was designed for the following future research objectives:

#### Better understand travel patterns and preferences

By examining the modes of transportation used, trip frequency, trip purpose, and travel time, we can identify trends and preferences among Hanoi’s residents. This information is crucial for designing policies that cater to the actual needs and desires of individual road users in Hanoi.

#### Assess the impact of socio-economic factors on travel behaviours

Understanding the influence of socio-economic factors such as occupation and living conditions on transportation choices will enable researchers to address potential equity issues in transport.

#### Evaluate public opinion on existing transportation infrastructure and services

Collecting feedback on the currently-used mobility modes will enable researchers to identify areas in need of improvement and prioritise future investments.

#### Gauge public perception of alternative transportation modes

UTM-Hanoi also has several interesting questions on alternative modes, e.g. the alternative modes of transport if motorbikes are banned in Hanoi, or a future vehicle that the respondent plans to purchase. This information can guide the development of initiatives that promote sustainable modes and reduce the dependency on motorbikes.

#### Identify barriers to adopting sustainable transportation modes

Investigating the factors that hinder the adoption of sustainable transportation options, such as safety concerns, lack of infrastructure, or insufficient public transit coverage, will enable the creation of context-specific, evidence-based transportation plans that address the unique challenges faced by Hanoi and promote sustainable urban mobility.

#### Facilitate comparative analysis with other cities

By collecting data that is comparable to similar surveys conducted in other cities, particularly in the Global South, we can identify best practices and lessons learned to inform Hanoi’s transportation planning and contribute to broader knowledge sharing.

The survey data set is expected to inform research and policy discussions on the development of effective and sustainable urban transportation policies and planning in Hanoi, and potentially in other cities facing similar challenges in the Global South.

### Data collection

The data collection process consisted of two distinct phases: an initial pilot survey followed by a comprehensive full-scale survey. The pilot phase aimed to validate the integrity of the survey questions and was initially implemented as a face-to-face interview by personnel and students from Vietnam National University (VNU) University of Science, utilising a data collection and analysis software known as Kobo Toolbox (https://www.kobotoolbox.org/). While the questions were originally designed to be bilingual (in English and Vietnamese), the limitations in language proficiency among the surveyors necessitated the exclusive use of the Vietnamese version. The pilot phase garnered approximately 1,500 responses before it was abruptly halted due to the COVID-19 pandemic, which rendered further data collection both impractical and ethically untenable. However, insights gleaned from this preliminary round contributed to refining the survey questions for the subsequent full-scale survey.

Upon the relaxation of pandemic containment measures in 2021 and the reestablishment of typical travel behaviours, the full survey was executed in a telephone-only format by the Vietnam General Statistics Office, Ministry of Planning and Investment. Owing to their extensive experience in conducting the Vietnam Census, the Ministry was well-equipped to secure a representative sample via telephone interviews. The original survey was truncated to better suit the telephone interview format. Utilising their comprehensive database of household contact information, a single family member was targeted for the survey, ensuring a high response rate. The survey aimed to capture data from a random 2.5% of households residing in central districts of Hanoi, as these regions would be most affected by the prospective motorbike ban. In cases where a household was unreachable, an alternative random household within the targeted region was selected. The final data set achieved a coverage of 2.51% of the total population for the four central districts in Hanoi, namely Hoan Kiem, Ba Dinh, Hai Ba Trung and Dong Da, with a sample size of *N* = 26,106 responses. Please note that the samples from the pilot study, as well as some incomplete samples from the full survey (that we could not validate) are not included in the dataset presented in this paper.

### Survey design

The UTM-Hanoi survey, that has resulted in the UTM-Hanoi^21^ dataset, was designed to elicit Hanoi residents’ preferences and behaviours related to transportation modes and policies. It began with general questions capturing respondents’ demographics like age, gender, occupation, and residential details. These personal attributes allow analysis of how opinions vary across different population groups.

A key component was a stated preference experiment examining a potential future ban on motorbikes in central Hanoi districts. First, respondents indicated their awareness and agreement with this proposed policy. They were then asked which alternate mode they would opt for if motorbikes were indeed prohibited. The options included cars, electric bikes, conventional bicycles, taxis, buses, light rail, and walking - representing the range of substitutes feasible in Hanoi.

Follow up questions had respondents select factors influencing their choices, like convenience, affordability, parking availability, and environmental friendliness. An initial pilot survey informed the categories and dominant options provided. This revealed preference experiment empirically quantifies mode shifting behaviours under the hypothetical ban scenario to evaluate likely policy impacts.

The categories for the categorical variables in UTM-Hanoi were developed during the pilot phase of data collection (with 1,500 respondents), when interviewers could enter open-ended responses for these questions. In the full survey, common themes were extracted to define the categories. For instance, when defining the categories for ‘reasons to choose a vehicle’, the main categories that emerged were convenience, cost, safety, flexibility, comfort, parking availability, and other. Respondents could select multiple choices from this defined list in the final survey. By leveraging the exploratory pilot to identify the most salient categories, we were able to design a streamlined dominant options for the final survey.

### Ethical validation

The UTM-Hanoi survey has been approved by the VNU University of Science Review Board for ethical clearance in 2020. The data collection complies with VNU’s ethical regulations on public opinion survey. All respondents to the survey were informed of the aims and objectives of the study and consented to open publication of their data. All identifiable personal information such as home address are removed, and home geospatial coordinates have been aggregated to the district’s centroid.

## Data Records

The UTM-Hanoi survey data are available publicly in the Open Science Foundation repository ‘UTM Hanoi’^21^. Data have been anonymised and cleaned to ensure maximum utility and have no risk of revealing personal information. All the variables and data have been translated into English from their original Vietnamese format. A full data codebook are available at the Supplementary information section of this paper including the full variable name, answer choices and answer labels for each question.

The UTM-Hanoi dataset is a comprehensive collection of data focusing on three primary aspects: general personal information, transport behavior, and general opinions and behaviors related to transport in Hanoi, Vietnam.

The first group of variables, “General Info”, encompasses demographic and residential information about the respondents. This includes gender, age, occupation, working hours, duration of living at the current residence, house price and type, and living status. Additionally, it incorporates the quality of living conditions, family age distributions, and types of vehicles owned by the family.

The second group, “Transport Behaviour”, captures the transport patterns of the respondents. It details the purpose of the transport, vehicle used, reasons for choosing a particular vehicle, and the frequency of transport. Specifics such as travel time and frequency of transport provide an in-depth understanding of transport behaviour.

The third group, “General Opinions and Behaviours”, includes general frequency of using each type of vehicle, plans for future vehicle purchases, reasons for not planning to purchase specific vehicles, and the distance to the nearest public transport. It also records the opinions of respondents on different types of vehicles, their awareness of a proposed motorbike ban, their opinion on this ban, their preferred alternative vehicles if the ban were to be implemented, and their reasons for choosing these alternatives.

Finally, locational data of the respondent’s home address such as the area identifier, unique respondent identifier, commune name, district name, and geospatial coordinates of the district centroid are also included in the dataset.

## Technical Validation

### Data consistency

We use statistical analysis to evaluate the dataset’s logical consistency among related items. We adopt the Cronbach’s alpha, a popular statistical method to access the internal consistency and reliability of a multi-items dataset^22^. Here ‘items’ can be samples of the dataset. The test evaluates how closely a set of items are related as a group and measures reliability based on the inter-variable correlations. For a scale with *k* components, *α* is defined as:

1 $$\alpha =\frac{k}{k-1}\left(1-\frac{\sum ^{k}i=1{\sigma }^{2}{Y}_{i}}{{\sigma }_{X}^{2}}\right)$$

where ${\sigma }_{{Y}_{i}}^{2}$ is the variance of component *i*, and ${\sigma }_{X}^{2}$ is the variance of the total score^22^. Values of *α* range from 0 to 1, with higher values indicating greater internal consistency.

We choose all numeric variables, except the geospatial coordinates and ids variables, to estimate the Cronbach’s alpha statistic. Here each row embodies an individual respondent’s dataset, and each column represents a specific variable under investigation. The resulted Cronbach’s alpha statistic is 0.62, showing “acceptable” internal consistency among the samples in our dataset, providing evidence that survey responses are reliable^22^.

Outliers, or data points significantly deviating from the central trend, are then identified and counted. We adopt the z-score method, which measures the standard deviations a data point is away from the mean^23^.

2 $$z=\frac{x-\mu }{\sigma }$$

Where: - *z* represents the z-score. - *x* is the individual data point. - *μ* denotes the mean of the dataset. - *σ* is the standard deviation of the dataset.

We estimate the z-score for every data point using all numeric variables. Any data point exhibiting a z-score beyond a predetermined threshold (set at 3 in this study) for any of the numeric column is then considered an outlier. This returns 445 outlier rows within the 26,106 total samples, or 1.7% within the dataset. This is an acceptable count of outliers in our dataset.

### Data representativeness

The final technical data validation is by comparing the statistical distributions in our dataset with the publically available information in other publications, as well as in the official statistics from government agencies in Vietnam to demonstrate data representativeness.

#### Spatial distribution of respondents

**Fig. 1:**
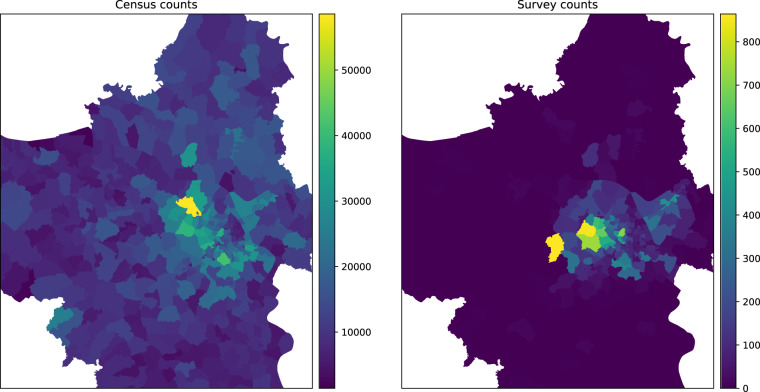
Map of counts of surveyed households compared to the distribution of households recorded in the census 2019 Vietnam census^20^.

The distribution of households selected for the survey in comparison to the distribution of households in the census is depicted in Fig. 1 (adopted from^20^). Although the sample size of our survey is substantial, it represents only a fraction of the Hanoi populace. However, it is noteworthy that the main urban centre of Hanoi is consistently sampled in our survey compared to the census data. This region is expected to be the most impacted by forthcoming transportation policies.

#### General statistics: gender, age and occupation

The general socio-demographic variables such as gender, age and occupation are the most fundamental variables in any individual survey. These variables are essential for promoting equity and accessibility, ensuring safety, designing targeted interventions, informing long-term planning, and guiding policy development for people.

**Fig. 2:**
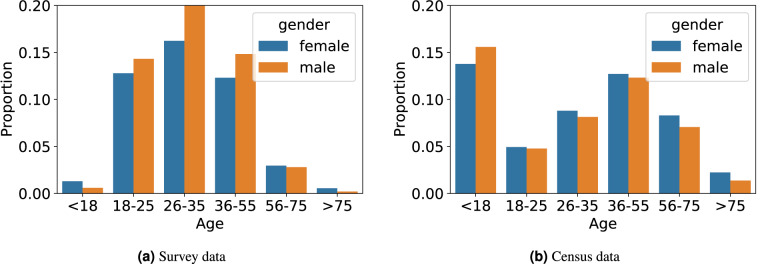
Age and gender distribution in Hanoi.

The gender distribution in UTM-Hanoi dataset is 54% male and 46% female^21^. Whilst this diverges from the expected gender distribution of 50/50, it is close to gender parity and similar to other travel survey in Hanoi^13,24^. Examining the age-gender distribution in more detail, Fig. 2 demonstrates a comparison of age and gender distributions in UTM-Hanoi and the census data. It may look like there is a large disparity between the two datasets at the upon a quick look, and this is because of the difference in data collection methods. While the Census dataset has been collected for all household members (thus it appears that the less than 18 years old is the largest age group), the UTM-Hanoi dataset has been collected by asking a single household member per household. This has led to an over-presentations of the 26 to 35 age group and males in UTM-Hanoi.

**Fig. 3:**
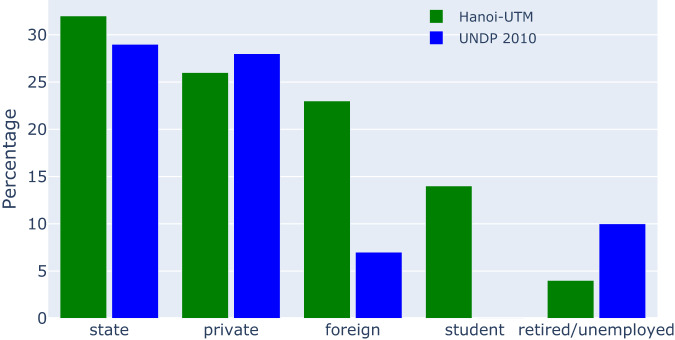
Distribution of occupation in the UTM-Hanoi dataset, in comparison to^25^.

We compare the occupation statistics from UTM-Hanoi^21^ to the Urban Poverty Survey data from the United Nations Development Programme (UNDP)^25^. Fig. 3 shows a relatively similar share of occupation in Hanoi, especially for the proportion of people who are working for a state-owned and private companies. The differences come from the fact that the UNDP dataset does not have “student” as an occupation (so they might be included as “unemployed”), and that UTM-Hanoi has more people listed as working for a “foreign” company. This might come from a different definition of a foreign company, or from the fact that from 2010 (UNDP dataset) to 2021 (UTM-Hanoi dataset), the share of foreign-invested or -based companies have increased significantly in Hanoi.

#### Trip purposes

**Fig. 4:**
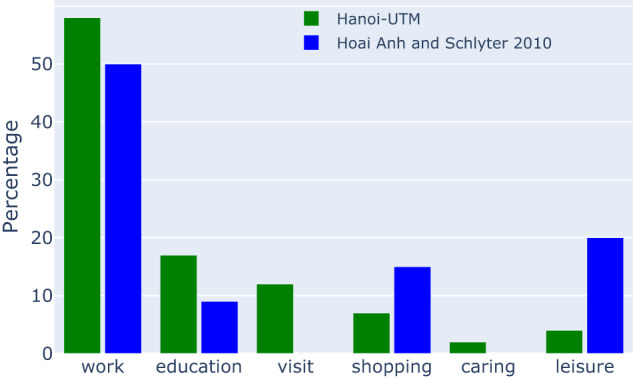
Distribution of trip purpose in the UTM-Hanoi dataset, in comparison to^26^.

**Fig. 5:**
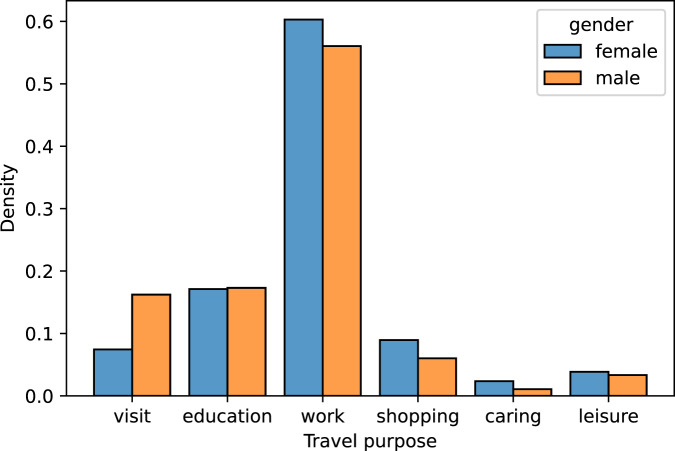
Distribution of trip purpose in the UTM-Hanoi dataset.

The trip purpose variable provides valuable insights into the travel behaviours and mobility needs of individuals. In UTM-Hanoi, the travel purpose variable is for the primary trip of the respondent, with 6 options to choose from (visit, education, work, shopping, caring and leisure)^21^. Fig. 4 compares the distribution of trip purpose in UTM-Hanoi with another study in literature that also provided similar statistics^26^. The differences in the way trip purposes were collected have led to a discrepancy in distributions between the two studies. UTM-Hanoi has slightly more commuting trips (work and education) and fewer recreational trips (shopping and leisure). This is because while^26^ asked for the “yesterday trip”, we asked for the “primary trip”. When we look deeper into the share of trip purposes by gender (Fig. 5), the distributions are also similar to^26^:

- Work trips are the most popular trip purpose for both male and female
- Females are making more shopping trips compared to males
- For “education” and “caring” trips, the distributions are relatively similar between the two genders.

#### Travel modes

**Fig. 6:**
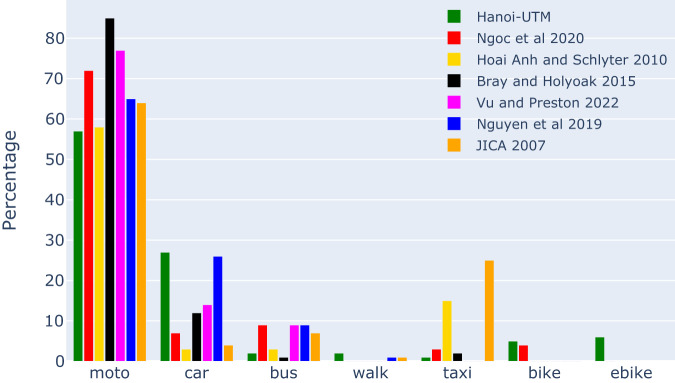
Modal share distributions in UTM-Hanoi dataset, in comparison with published studies.

Travel mode share is the proportion of total trips made by each mode of transport, such as private cars, motorbikes, buses or walking. This variable is essential to understand individual travel behaviour and choices, which in turn can inform transport planing and policy development. Existing studies reported different modal share for transportation modes in Hanoi, as illustrated in Fig. 6.

While it is widely known that the majority of people in Hanoi use motorbikes to travel, existing studies reported a wide range of modal share for motorbikes in Hanoi, with our UTM-Hanoi dataset^21^ reports just short of 60%, while^13^ reported more than 80%, and other studies^26–30^ reported a value in between 60% to 80%. The value in UTM-Hanoi is similar to studies that reported around 60% of motorbike modal share in Hanoi^26,29,30^. A possible reason for the discrepancy in literature are the differences in the way data are collected. In^13^, modal share data are collected by directly observing the traffic flow in Hanoi, while in other studies, including our own, the modal share comes from a travel survey. UTM-Hanoi, along with^29^, also reports the highest share of car usage in Hanoi at nearly 30%. In UTM-Hanoi, the interviewee is often the head of household, who is more likely to use the only car of the household, while other family members may use a different mode to travel. Nevertheless, UTM-Hanoi, along with many other studies^13,27–29^, reports a similar value for the total of private vehicle modal share (motorbikes and cars) of 90%, which is also consistent with a report by Hanoi Metropolitan Railway Management Board (MRB)^31^.

#### Vehicle ownership

**Fig. 7:**
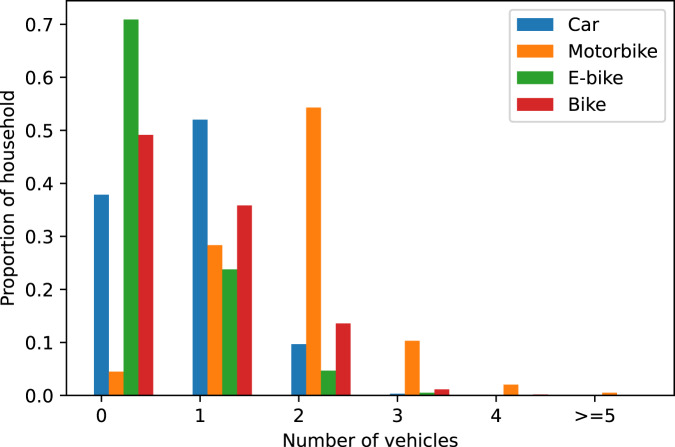
Modal share distributions in UTM-Hanoi dataset, in comparison with published studies.

Individual vehicle ownership data in households is useful for addressing the traffic congestion and safety problems in Hanoi. It can be used to identify hot spots of high private vehicle usage to prioritise public transport investments, promote active transport and implement demand management strategies. Figure 7 illustrates the vehicle ownership data for individual households in the UTM-Hanoi survey^21^. It confirms a well-known fact that most households in Hanoi own motorbikes, with 95% of all households owning at least one, and more than 20% owning more than two. This is somewhat consistent with statistics from a JICA project in 2005, which showed that there are 83% of households in Hanoi who own motorbikes, with 40% of them having more than 2^30^. Bray and Holyoak^13^ also stated that the majority of households in Hanoi had two or more motorbikes in their 2015 study. However, it is a concerning fact that the share of car ownership has increased dramatically from 2% in 2005^30^ to 8% in 2015^13^ and now to 60% in our dataset. An explaination for this may come from the fact that UTM-Hanoi is a survey focuses at the CBD area of Hanoi, which is a more wealthy area of the city.

#### Opinion on different modes of transport

**Fig. 8:**
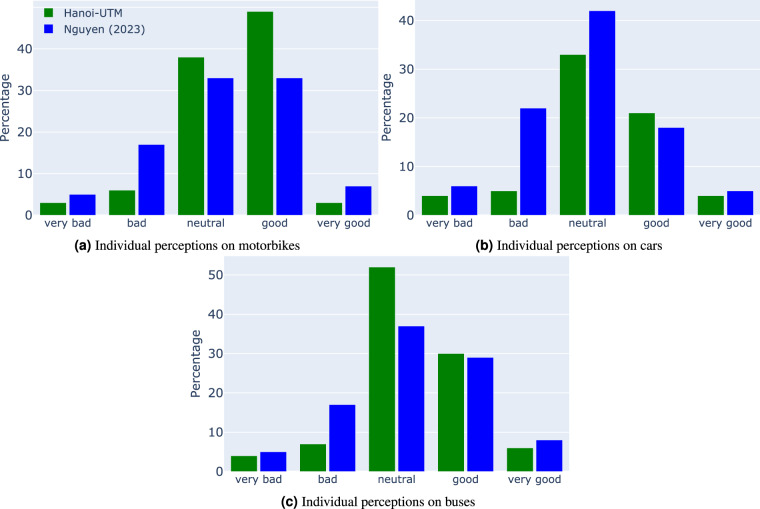
Reasons why travel modes are not chosen and not chosen.

Individual perceptions on transportation modes, and opinions on transportation policies (such as the motorbike ban) are key in the UTM-Hanoi dataset^21^. UTM-Hanoi provides rich information about individual opinions on different travel modes in Hanoi, with responses varying on a 5-point Likert scale from “very bad” to “very good”. However, it is challenging to validate these because individual perception studies are rare everywhere, let alone in Hanoi. We can find only another recent study^32^ that talks about individual perceptions on transportation modes in Hanoi. Figure 8 shows the share of opinion on the four main modes of transport (motorcycles, cars and buses, cars). The first pattern we can identify is that the majority of people have a neutral view on the main modes of transport, and there are only a very small proportion of people who has strong views (“very good” or “very bad”) on these modes on Table 1.

**Table 1:** Main survey questions.

Group	Questions (selected)
General info	Age, gender, location, occupation,
Living conditions	Living duration, property type, status, home ownership, water quality, open space, noise, school access, market access, hospital access, bank access, security, leisure access, transport satisfaction
Family composition and vehicle ownership	Age <18, age 18–25, age 26–35, age 36–55, age 56–75, age 75+, family car, family motorbike, family e-bike, family bike
Primary trip	Origin, destination, travel purpose, mode choice, the reason for mode choice, travel time, frequency per day, frequency per week, frequency per month,
Mode choice	Frequency usage of a car, motorbike, e-bike, bike, bus; future purchase; reason not to buy: car, motorbike, e-bike, bike; distance to public transport; opinion of mode: car, motorbike, e-bike, bike, taxi, bus
Motorbike ban	Awareness of the potential motorbike ban, opinion, alternative vehicle: car, e-bike, bike, taxi, bus, light rail, taxi, walk; reason for vehicle ban: convenience, cost, parking, other

### Supplementary information

Supplementary Information

## Data Availability

The code used for exploratory data analysis, validation and visualization in this study is openly available for access and use. The codebase, which includes Jupyter Notebooks, Python scripts, and relevant libraries, is hosted on a public GitHub repository (https://github.com/Urban-Analytics/UTM-Hanoi). The code is distributed under the MIT License, allowing for modification, distribution, and reuse, as long as proper credit is given to the original authors and the license terms are followed.

## References

[1] Van, T. H., Schmoecker, J.-D. & Fujii, S. Upgrading from motorbikes to cars: Simulation of current and future traffic conditions in ho chi minh city. In *Proceedings of the Eastern Asia Society for Transportation Studies Vol. 7 (The 8th International Conference of Eastern Asia Society for Transportation Studies, 2009)*, 335–335 (Eastern Asia Society for Transportation Studies, 2009).

[2] Hansen, A. *Driving Doi Moi*, 486–501, first edn (Routledge, London, 2022).

[3] Hansen, A. Hanoi on wheels: emerging automobility in the land of the motorbike. *Mobilities* 1–18, 10.1080/17450101.2016.1156425 (2016).

[4] Truitt A (2008). On the back of a motorbike: Middle-class mobility in ho chi minh city, vietnam. American ethnologist.

[5] Turner S (2020). Informal motorbike taxi drivers and mobility injustice on hanoi’s streets. negotiating the curve of a new narrative. Journal of transport geography.

[6] Pham TXT, Nguyen NT, Duong LBT (2021). Hierarchy-attribute decision making regarding public buses and private motorbikes: a case study in ho chi minh city, vietnam. Public Transport.

[7] Thuy PC, Kameda T, Toriba A, Tang N, Hayakawa K (2012). Characteristics of Atmospheric Polycyclic Aromatic Hydrocarbons and Nitropolycyclic Aromatic Hydrocarbons in Hanoi-Vietnam, as a Typical Motorbike City. Polycyclic Aromatic Compounds.

[8] Truong LT, Kieu L-M, Vu TA (2016). Spatiotemporal and random parameter panel data models of traffic crash fatalities in Vietnam. Accident Analysis & Prevention.

[9] Ly B-T (2020). Characteristics of roadside volatile organic compounds in an urban area dominated by gasoline vehicles, a case study in Hanoi. Chemosphere.

[10] Chou, C.-C., Yoh, K., Inoi, H., Yamaguchi, T. & Doi, K. Effectiveness evaluation on cross-sector collaborative education programs for traffic safety toward sustainable motorcycle culture in vietnam. *IATSS research* (2022).

[11] Tung, A. K.-D. Hanoi prepares conditions to ban motorcycles by 2030. *Hanoi Times* (2020).

[12] Hansen A (2017). Transport in transition: Doi moi and the consumption of cars and motorbikes in Hanoi. Journal of Consumer Culture.

[13] Bray, D. & Holyoak, N. Motorcycles in Developing Asian Cities: A Case Study of Hanoi (2015).

[14] Calvo-Poyo F, Medialdea A, Ferri-García R (2020). Citizens’ opinion about investment in public transport projects in cities. International Journal of Sustainable Transportation.

[15] Luke R, Heyns G (2013). Public transport policy and performance: The results of a south african public opinion poll. Journal of Transport and Supply Chain Management.

[16] Hanoi People’s Council. 5953/qd-ubnd on approving the scheme “strengthening the management of road transport means to reduce traffic congestion and environmental pollution in hanoi city, the period of 2017–2020 vision 2030” (2017).

[17] Comber, A. *et al*. Multiscale Geographically Weighted Discriminant Analysis. *GIScience 2021 Short Paper Proceedings. 11th International Conference on Geographic Information Science. September 27-30* **2021. Poznań**, Poland (Online), 10.25436/E2PP4F (2021).

[18] Comber A (2022). Handling the MAUP: Methods for identifying appropriate scales of aggregation based on measures on spatial and non-spatial variance. AGILE: GIScience Series.

[19] Malleson, N. *et al*. Urban Data Science for Sustainable Transport Policies in Emerging Economies. *GIScience 2021 Short Paper Proceedings. 11th International Conference on Geographic Information Science. September 27-30* **2021. Poznań**, 10.25436/E28G6D (2021).

[20] Kieu M (2023). Factors affecting perceptions in transport–a deep dive into the motorbike ban in hanoi, vietnam. Case Studies on Transport Policy.

[21] Malleson N (2023). OSF.

[22] Cronbach LJ (1951). Coefficient alpha and the internal structure of tests. psychometrika.

[23] Shiffler RE (1988). Maximum z scores and outliers. The American Statistician.

[24] Hoang-Tung N (2022). Ride-hailing service adoption and local context in motorcycle-based societies: case study in hanoi, vietnam. Sustainability.

[25] UNDP. Urban poverty assessment in ha noi and ho chi minh city (2010).

[26] Tran HA, Schlyter A (2010). Gender and class in urban transport: the cases of xian and hanoi. Environment and Urbanization.

[27] Ngoc, A. M., Nishiuchi, H., Van Truong, N. & Huyen, L. T. A comparative study on travel mode share, emission, and safety in five vietnamese cities. *International Journal of Intelligent Transportation Systems Research* 1–13 (2022).

[28] Vu T, Preston J (2022). A comparative economic assessment of urban transport infrastructure options in low-and middle-income countries. Transportation Research Part A: Policy and Practice.

[29] Nguyen TT, Nguyen HTA, Xuan C, Fujiwara A (2019). Income-based fare orientation in urban public transportation services in developing countries: A case study in hanoi, vietnam. Journal of the Eastern Asia Society for Transportation Studies.

[30] Iwata, S. *et al*. The comprehensive urban development programme in hanoi capital city of the socialist republic of vietnam (haidep). *Final report, JICA, Hanoi* (2007).

[31] (MRB), H. M. R. M. B. Hanoi metro: A sustainable public transport for densely populated cities (2018).

[32] Hoang-Tung, N. Recognizing the involvement of satisfaction in nurturing habits of travel mode use. *International Journal of Intelligent Transportation Systems Research* 1–17 (2023).

