# Supplementary material for: An open dataset on individual perceptions of transport policies
Source: Sci Data. 2024 Jan 22;11:104. doi: 10.1038/s41597-024-02950-9 (PMC10803299; doi:10.1038/s41597-024-02950-9)
Supplement: Supplementary file 1 — Supplementary Information [file 41597_2024_2950_MOESM1_ESM.pdf]

Supplementary materials for: An open data on individual perceptions of transport policies

ABSTRACT

This is the supplementary materials for: An open data on individual perceptions of transport policies

Data codebook

Table 1. Data Description of UTM-Hanoi Dataset

Variable	Data Type	Description
	Group 1: General Info	
Gender	Categorical	Gender of the respondent
Age	Numerical	Age of the respondent
Your_occupation	Categorical	Occupation of the respondent
Working_time	Numerical	Working hours per week
Living_duration	Numerical	Years living in current house
House_price	Numerical	Price of the house
House_type	Categorical	Type of house (apartment, detached, etc.)
Living_status	Categorical	Living status (alone, with family, etc.)
House_ownership	Categorical	House ownership status
Living_quality	Numerical	Ratings of different living quality aspects
Family_ages	Numerical	Age distribution within family members
Family_vehicle	Categorical	Types of vehicle owned by the family
	Group 2: Transport Behaviour	
Transport_purpose	Categorical	Purpose of the transport
Transport_vehicle	Categorical	Vehicle used for transport
Reason_to_choose	Categorical	Reasons to choose the specific vehicle
Travel_time	Numerical	Travel time in minutes
Frequency	Categorical	Frequency of the transport
	Group 3: General Opinions and Behaviours	
Frequency_general	Categorical	General frequency of using each type of vehicle
Future_vehicle	Categorical	Type of vehicle planning to purchase in the future
Reason_not_to_buy	Categorical	Reasons for not planning to purchase a specific vehicle
Distance_to_public	Numerical	Distance to the nearest public transport
Opinion_vehicle	Categorical	Opinions on different types of vehicle
Aware_of_ban	Binary	Awareness of the proposed motorbike ban
Opinion_on_ban	Categorical	Opinion on the proposed motorbike ban
Alternative_vehicle	Categorical	Preferred alternative vehicle if motorbikes are banned
Reason_to_choose_vehicle_ban	Categorical	Reasons to choose the alternative vehicle
AreaID	Categorical	Area identifier
Code	Categorical	Unique identifier for each respondent
Comu_name	Categorical	Name of the commune
Dist_name	Categorical	Name of the district
District_centroid	Numerical	Coordinates of the district centroid
